# Checklist of the vascular flora of the Sunda-Sahul Convergence Zone

**DOI:** 10.3897/BDJ.8.e51094

**Published:** 2020-05-18

**Authors:** Elizabeth M. Joyce, Kevin R. Thiele, Ferry J.W. Slik, Darren M. Crayn

**Affiliations:** 1 Australian Tropical Herbarium, James Cook University, Cairns, 4870, Australia Australian Tropical Herbarium, James Cook University Cairns, 4870 Australia; 2 College of Science and Engineering, James Cook University, Cairns, 4870, Australia College of Science and Engineering, James Cook University Cairns, 4870 Australia; 3 Centre for Tropical Environmental Sustainability Science, James Cook University, Cairns, 4870, Australia Centre for Tropical Environmental Sustainability Science, James Cook University Cairns, 4870 Australia; 4 School of Biological Sciences, The University of Western Australia, Crawley, 6009, Australia School of Biological Sciences, The University of Western Australia Crawley, 6009 Australia; 5 Faculty of Science, Department of Environmental and Life Sciences, Universiti Brunei Darussalam, Gadong BE1410, Brunei Faculty of Science, Department of Environmental and Life Sciences, Universiti Brunei Darussalam Gadong BE1410 Brunei

**Keywords:** Southeast Asia, Australia, Wallacea, biogeography, exchange, species list, diversity

## Abstract

**Background**

The Sunda-Sahul Convergence Zone, defined here as the area comprising Australia, New Guinea, and Southeast Asia (Indonesia to Myanmar), straddles the Sunda and Sahul continental shelves and is one of the most biogeographically famous and important regions in the world. Floristically, it is thought to harbour a large amount of the world’s diversity. Despite the importance of the area, a checklist of the flora has never before been published. Here we present the first working checklist of vascular plants for the Sunda-Sahul Convergence Zone. The list was compiled from 24 flora volumes, online databases and unpublished plot data. Taxonomic nomenclature was updated, and each species was coded into nested biogeographic regions. The list includes 60,415 species in 5,135 genera and 363 families of vascular plants.

**New information**

This is the first species-level checklist of the region and presents an updated census of the region’s floristic biodiversity. The checklist confirms that species richness of the SSCZ is comparable to that of the Neotropics, and highlights areas in need of further documentation and taxonomic work. This checklist provides a novel dataset for studying floristic ecology and evolution in this biogeographically important region of very high global biodiversity.

## Introduction

**Figure 1.**
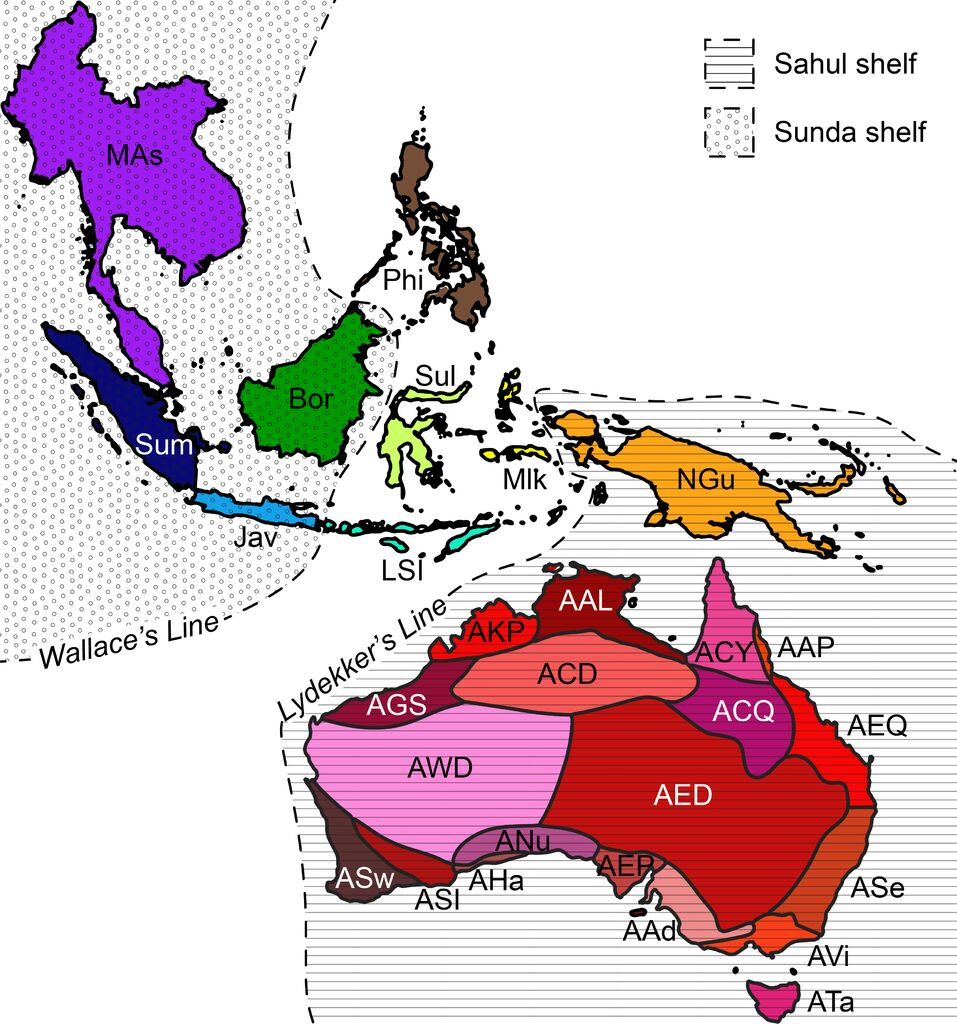
Map showing the continental shelf, island area and Australian phytogeographic subregions (Ebach et al. 2015) that species were coded to in the SSCZ vascular plant checklist. MAS, Mainland Asia; Sum, Sumatra; Bor, Borneo; Jav, Java; Phi, Philippines; Sul, Sulawesi; LSI, Lesser Sunda Islands; Mlk, Maluku Islands; NGu, New Guinea; AKP, Kimberley Plateau; AAL, Arnhem Land; ACY, Cape York Peninsula; AAP, Atherton Plateau; AGS, Great Sandy Desert Interzone; ACD, Central Desert; ACQ, Central Queensland; AEQ, Eastern Queensland; AWD, Western Desert; AED, Eastern Desert; ASe, Southeastern; ASw, Southwestern; ASI, Southwest Interzone; AHa, Hampton; ANu, Nullarbor; AEP, Eyre Peninsula; AAd, Adelaide; AVi, Victoria; ATa, Tasmania.

The Sahul continental shelf comprises Australia and the island of New Guinea, while the Sunda continental shelf comprises mainland Southeast Asia (Myanmar, Cambodia, Vietnam, Laos, Thailand, Peninsular Malaysia) and Indonesia west of Wallace’s Line. Between the Sunda and Sahul shelves lies Wallacea, a biogeographic region composed of a complex conglomerate of continental fragments and island arcs of varied origins (Hall 2012). Wallacea encompasses the Philippines, Timor-Leste, and parts of Indonesia (Fig. 1).

The Sunda and Sahul shelves converged approximately 25 Mya, following the rifting of Sahul from Antarctica approximately 45 Mya, its northward drift, and its contact with Wallacea and Sunda at the Oligocene-Miocene boundary (Hall 2002, Zahirovic et al. 2016). This collision facilitated the exchange of biota that had been evolving in isolation for at least 20 My (Crayn et al. 2015, Sniderman and Jordan 2011, Kooyman et al. 2019). The Sunda-Sahul biotic exchange provides a natural experiment to study evolution and biogeography, and has fascinated scientists since the time of Alfred Wallace (Wallace 1860). However, despite considerable interest, study and speculation on the biogeography of the region, much remains to be understood regarding which taxa were exchanged, when they were exchanged and the processes by which this occurred.

A plethora of terms for areas and the floristic exchange that occurred between them has accumulated due to the longstanding biogeographic interest in the region. Parts of the area of focus have been variously referred to as the ‘Indo-Australian archipelago’ (Lohman et al. 2011), the ‘Southeast Asian-Australian region’ (Metcalfe 2001, Morley 2002), the ‘Malay archipelago’ (Wallace 1860, Hall 2017), ‘Malesia’ (Steenis 1979, [Bibr B5511662][Bibr B5511568]), ‘Papuasia’ (Brummitt et al. 2001) and ‘Australasia’ (Heads 2014), based on floristic or political boundaries. Richardson et al. 2012 referred to the floristic exchange as the ‘Malesian Floristic Interchange’, while Crayn et al. 2015 named it the ‘Sunda-Sahul Floristic Exchange’. To adequately describe the geological history of the area and the area likely to be affected by the exchange of flora between the Sunda and Sahul shelves, we herein introduce the term “The Sunda-Sahul Convergence Zone” (SSCZ) to describe the area of focus. This area comprises the entire Sahul shelf (Australia and New Guinea), Wallacea (the Moluccas, Sulawesi, the Philippines and the Lesser Sunda Islands), and the Sunda shelf (Borneo, Sumatra, Java, and mainland Asia to Myanmar) (Fig. 1). Furthermore, we here adopt the term “the Sunda-Sahul floristic exchange” (Crayn et al. 2015), as it most accurately describes the exchange of flora upon the convergence of the two continental shelves.

The SSCZ is thought to harbour a significant proportion of the world’s biodiversity, yet much of this biodiversity remains to be documented. Four of the world’s ‘biodiversity hotspots’ occur in the SSCZ - the Philippines, Sundaland, Wallacea and Southwest Australia (Myers et al. 2000). The SSCZ also contains the ‘major wilderness area’ of New Guinea—an area thought to have a high degree of endemism and diversity but that is not currently considered to be under threat (Mittermeier et al. 1998). However, despite the clear importance of the region and its biodiversity, its flora has never been documented as a whole.

Checklists for some parts of the SSCZ exist, but their completeness, currency and availability differ. Some are in hard copy form in books or journals (e.g. [Bibr B5511850][Bibr B5511649]), some are online (e.g. Pelser et al. 2011), and some are unpublished (e.g. Slik 2018). Many are not publicly available or are out of print. The hard copy formats are static and not up-to-date with respect to new taxonomic discoveries or revised taxonomic concepts. The taxonomies used in these lists also often differ, mostly due to their different publication dates, making comparison of taxa between regions difficult.

The flora of the SSCZ must be documented for it to be studied, analysed and conserved. Documentation is also necessary to appreciate the significance of the SSCZ flora on a global scale, and to further our understanding of its ecology and evolution. Therefore, we provide here for the first time a digital, comprehensive, updateable and publicly available dataset of vascular plants for the SSCZ.

## Materials and methods

A checklist of the vascular flora of the SSCZ was compiled from 26 sources including flora volumes, published checklists and databases, and unpublished plot data and checklists (Table 1). For areas where these data were inadequate or lacking, they were supplemented with herbarium specimen-based occurrence records from GBIF.

For floras and checklists in hardcopy, scanned copies were converted into plain text with Optical Character Recognition (OCR). Scientific binomial names were extracted from the plain text documents using the Global Names Recognition and Discovery Service v.0.8.5 (Marine Biological Laboratory 2016; http://gnrd.globalnames.org/name_finder). All names were manually checked for accuracy against the original source and corrected as necessary. Species noted to be non-native in floras and checklists were removed.

The taxonomic status of names from each source was checked using the Taxonomic Name Resolution Service v.4.0 (iPlant Collaborative 2019, Boyle et al. 2013; http://tnrs.iplantcollaborative.org/TNRSapp.html). Names were processed in the “Perform Name Resolution” mode allowing partial matches with a minimum threshold of 0.05. Sources selected to check names were Tropicos.org (Missouri Botanical Garden 2019), the Global Compositae Checklist (Flann 2009), the International Legume Database and Information Service (ILDIS 2018), The Plant List (The Plant List 2013) and The PLANTS Database (USDA and NRCS 2019). Tropicos.org was selected as the family classification source as the nomenclature is known to be actively updated and current. All matches were manually inspected for anomalies and corrected where necessary. For synonymised names, the currently accepted name according to the Taxonomic Name Resolution Service v.4.0 was included in the checklist. Names returning the taxonomic status of “No Opinion” were also included; these names are awaiting assessment by name-checking sources, but for our purposes were assumed to be accepted to ensure they were not prematurely excluded from the final list. Phrase names, manuscript names, hybrids and infraspecific taxa were omitted to increase the likelihood that the species included are recognised internationally. All species names were then classified to a major group: Angiosperms, Fern and fern allies and Gymnosperms.

Species from each source were coded according to their country, island group and continental shelf (Fig. 1). Australian species from the Australian Plant Census (APC; Centre for Australian National Biodiversity Research and Council of Heads of Australasian Herbaria 2017) were further coded by their occurrence in the Australian Bioregionalisation Atlas phytogeographic subregions (Ebach et al. 2015). This was done by downloading occurrence data for every species on the APC from GBIF using the rgbif v.1.3.0 package in R (Chamberlain et al. 2019). Occurrence data were cleaned to include only herbarium records from after 1960 with a geospatial coordinate uncertainty of less than 25 km. Geospatial coordinates for each species were then coded into phytogeographic subregion polygons using the speciesgeocodeR package in R (Töpel et al. 2017). Phytogeographic subregion polygons were modified from the study of González-Orozco et al. (2014) and Ebach et al. 2015 to incorporate the administrative boundary of Australia so that offshore islands (e.g. the Torres Strait Islands) were included. Species occurrences in the Australian external territories of Norfolk Island, Australian Antarctic Territory, Heard and McDonald Islands, Coral Sea Islands, Christmas Island, Ashmore and Cartier Islands were excluded. This process was repeated for the Australian Orchidaceae list from (Royal Botanic Gardens, Kew 2019).

Source lists were then merged and duplicates removed. Consistency in family classification was checked.

## Checklist of the vascular flora of the SSCZ

The checklist of the vascular flora of the SSCZ (Suppl. material 1) has been stored in the Research Data JCU Tropical Data Hub (DOI: http://dx.doi.org/10.25903/5ea0dd85f8ea9). Updated versions of the checklist can be accessed via www.ath.org.au/australian-tropical-herbarium/datasets.

## Analysis

A total of 60,415 species in 5,135 genera and 363 families of vascular plants are recorded in the Sunda-Sahul Convergence Zone. The number of taxa in each island group and continental shelf are summarised in Table 2.

## Discussion

Here we present the first comprehensive species checklist of native vascular plants for the Sunda-Sahul Convergence Zone, comprising 60,415 species. An estimated 374,000 vascular plant species are known globally (Christenhusz and Byng 2016); thus, our checklist indicates that the SSCZ harbours at least 16.2% of all known vascular plant species. Considering the land area of the SSCZ (c. 12,215,000 km^2^, c. 8% of the global land area), our estimated diversity for the region is substantially higher than the global average number of species per unit area.

It has long been assumed that the Neotropics are more species-rich than the Southeast Asian tropics. However, our findings suggest that floristic richness in the SSCZ is comparable to that of the Neotropical ecozone (*sensu* Schultz 2005) which extends from central Mexico to southern Brazil, a latitudinal range similar to that of the SSCZ. Approximately 90,000–110,000 seed plant species are estimated to occur in the Neotropical ecozone (Antonelli and Sanmartín 2011), an average of 0.0062 seed plant species per km^2^. By comparison, the SSCZ has an average of 0.0047 seed plant species per km^2^. The average for the whole SSCZ is lowered by the inclusion of Australia, which mostly comprises savannah and arid biomes known to have relatively low floristic richness. Excluding Australia, the SSCZ has 0.0088 vascular plant species per km^2^, which is slightly higher than the richness of seed plants in the Neotropical ecozone. Similar species richness between the Southeast Asian tropics and Neotropics was also recently reported for tropical tree species by Slik et al. (2015).

**Figure 2.**
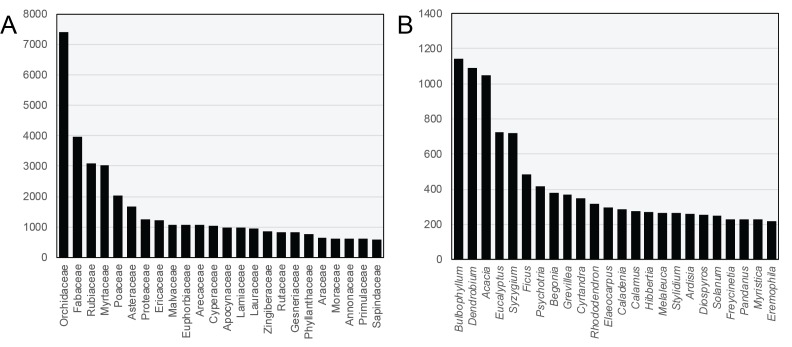
25 most species-rich families (A) and genera (B) in the SSCZ

The five most species-rich families in the region are Orchidaceae, Fabaceae, Rubiaceae, Myrtaceae and Poaceae (Fig. 2). The most species-rich genera are *Bulbophyllum* and *Dendrobium* (Orchidaceae), *Acacia* (Fabaceae), and *Eucalyptus* and *Syzygium* (Myrtaceae). Orchidaceae, Fabaceae and Poaceae are some of the most species-rich plant families on Earth, and thus their predominance in the region is expected. The pattern of the most diverse families in the SSCZ is similar to that of the Amazon; however, the Amazon includes a high number of Melastomataceae species, fewer Proteaceae species and Ericaceae species, and the SSCZ has ten times the number of orchid species than the Amazon (Cardoso et al. 2017). Only one genus - *Psychotria* (Rubiaceae) - exhibits a similarly high diversity in the Amazon and the SSCZ, reflecting the independent evolutionary histories of these tropical floras.

It must be emphasised that this is a working checklist of vascular plants; the aim was to compile current knowledge of floristic distribution across the region in an objective and systematic way, and to publish it in a digital, updateable format. Some inevitable errors in taxonomy and distribution will be present in the dataset, reflective of errors in the taxonomic backbones used to standardise nomenclature across sources. These will be corrected over time through consultation with group experts and other regional flora projects, and updated versions of the checklists will be released. The number of errors is likely to be small and unlikely to invalidate results of analyses based on this checklist, given the size of the dataset and the diversity and reliability of source lists. The dataset also almost certainly under-represents actual floristic diversity in the region. Many areas within the SSCZ are underexplored, and therefore have a biodiversity that is not accurately documented. This is particularly the case in many parts of Indonesia, New Guinea, Cambodia and Vietnam. Additional taxonomic work is urgently needed to fully understand and refine species boundaries in poorly known groups. This is challenging in an area so geographically and politically diverse, and is particularly important given current and emerging threats to the biodiversity of the region (Myers et al. 2000). Given the digital format of this checklist, the checklist is able to be updated as new discoveries are made and taxonomies are revised. It provides a baseline overview of current knowledge of the regional flora for biodiversity research, which can be built on and refined over time.

The checklist is provided as a resource for scientists studying the biodiversity, evolution, biogeography and ecology of this region. Questions generated from this list include the following:

- Which taxa have been exchanged between the Sunda and Sahul shelves?
- Is there a difference between functional traits of plants between different islands, and what could be driving this?
- Where are the most diverse areas that should be considered for conservation priority?
- What are the environmental correlates of variation in floristic composition across the region?

The list of vascular flora of the SSCZ also offers opportunities to build a regional database of plant traits for ecological and evolutionary research. Ultimately, we hope that this checklist will provide a resource to enable researchers to generate and test biogeographic, ecological and evolutionary hypotheses in this globally megadiverse and biogeographically important region.

## Supplementary Material

7C76A6F4-9BFB-5552-8962-0FB26148AE7A 10.3897/BDJ.8.e51094.suppl1 Supplementary material 1 Checklist of Vascular Flora of the Sunda-Sahul Convergence Zone Data type Species checklist Brief description A list of every species of vascular plant in the Sunda-Sahul Convergence Zone, coded by country, island group and continental shelf.
Island group and country codes:
Bor = Borneo (whole island); Bru = Brunei; Cam = Cambodia; IBo = Indonesian Borneo; ING = Indonesian New Guinea; Jav = Java; Lao = Laos; LSI = Lesser Sunda Islands (including Timor); Ind = Indonesia; MAs = Mainland Asia; Mlk = Maluku Islands; Mly = Malaysia; Myn = Myanmar; NGu = New Guinea (whole island); PNG = Papua New Guinea; Tha = Thailand; Vie = Vietnam; Sin = Singapore; SSa = Sabah and Sarawak; Sul = Sulawesi; Sum = Sumatra; Tim = Timor
Continental shelf codes:
Sah = Sahul; Sun = Sunda; Wal = Wallacea File: oo_377596.xlsx https://binary.pensoft.net/file/377596 E.M. Joyce, K.R. Thiele, J.W.F. Slik, D.M. Crayn

## Figures and Tables

**Table 1.** Sources of species names for the Sunda-Sahul Convergence Zone vascular plant checklist.

**Island area**	**Country**	**Shelf**	**Source**
Australia	Australia	Sahul	Centre for Australian National Biodiversity Research and Council of Heads of Australasian Herbaria 2017
Australia	Australia	Sahul	Royal Botanic Gardens, Kew 2019
Borneo	Brunei, Malaysia and Indonesia	Sunda	GBIF 2017b
Borneo	Indonesia	Sunda	Slik 2018, XMalesia 2019
Borneo	Brunei	Sunda	Slik 2018, Slik 2019
Borneo	Malaysia	Sunda	Soepadmo and Wong 1995, Soepadmo et al. 1996; Soepadmo and Saw 2000, Soepadmo et al. 2002; Soepadmo et al. 2004, Soepadmo et al. 2007
Java	Indonesia	Sunda	GBIF 2017e, Slik 2018
Lesser Sunda Islands	Indonesia, Timor-Leste	Wallacea	Monk et al. 1997, GBIF 2017c, XMalesia 2019
Lesser Sunda Islands	Indonesia	Wallacea	XMalesia 2019
Mainland Asia	Malaysia	Sunda	Forest Research Institute Malaysia 2017, Slik 2018, Botanic Gardens Conservation International 2019
Mainland Asia	Cambodia	Sunda	GBIF 2017f, Slik 2018, Botanic Gardens Conservation International 2019
Mainland Asia	Laos	Sunda	Newman et al. 2007, GBIF 2017g, Slik 2018, Botanic Gardens Conservation International 2019
Mainland Asia	Myanmar	Sunda	Kress et al. 2003, GBIF 2017h
Mainland Asia	Thailand	Sunda	Chayamarit et al. 2014, GBIF 2017a, Slik 2018, Botanic Gardens Conservation International 2019
Mainland Asia	Vietnam	Sunda	GBIF 2017i, Slik 2018, Botanic Gardens Conservation International 2019
Mainland Asia	Singapore	Sunda	Chong et al. 2009, Slik 2018
Maluku Islands	Indonesia	Wallacea	Monk et al. 1997, GBIF 2017j, XMalesia 2019
New Guinea	Papua New Guinea, Indonesia	Sahul	Botanical Research Institute of Texas 2017, James 2017, GBIF 2017m
New Guinea	Papua New Guinea	Sahul	Womersley 1978, Henty 1981, Conn 1995, Slik 2018
New Guinea	Indonesia	Sahul	Slik 2018, XMalesia 2019
Philippines	Philippines	Wallacea	Pelser et al. 2011, GBIF 2017k, Slik 2018
Sulawesi	Indonesia	Wallacea	Kessler et al. 2002, GBIF 2017l, Slik 2018
Sumatra	Indonesia	Sunda	GBIF 2017d, Slik 2018

**Table 2.** Summary of number of species, genera and families in each island group and continental shelf

**Shelf**	**Island group**	**No. species**	**No. genera**	**No. families**
Sahul	Australia	20430	2188	258
	New Guinea	15765	2231	275
	Sahul total	34229	3252	310
Wallacea	Lesser Sunda Islands	1488	793	169
	Maluku Islands	2590	1116	208
	Philippines	10212	2023	263
	Sulawesi	3094	1188	212
	Wallacea total	12802	2308	272
Sunda	Borneo	8458	1641	235
	Java	1676	837	180
	Mainland Asia	17861	3189	302
	Sumatra	2941	1035	202
	Sunda total	23331	3499	309
SSCZ	SSCZ total	60526	5135	363
